# 

**Published:** 1843-02

**Authors:** 


### THE WESTERN JOURNAL OF MEDICINE AND SURGERY:

EDITED BY

DANIEL DRAKE, M. D.

AND

LUNSFORD P. YANDELL, M. D.

PROFESSORS IN THE LOUISVILLE MEDICAL INSTITUTE,

AND

THOMAS W. COLESCOTT, M. D.

NO. XXXVII.—FEBRUARY, 1843.

VOL. VII.—NO. II.

LOUISVILLE, KY.

PUBLISHED MONTHLY, BY PRENTICE AND WEISSINGER,

PROPRIETORS AND PRINTERS.

1843.

*This Number contains 4 sheets—Postage under* 100 *miles* 6 *cents, over* 100 *miles* 10 *cents*

